# The Temporal Resolution Needed for Speech Intelligibility Assessed with Mosaic Speech: Effects of Block Duration, Age, and Word Familiarity

**DOI:** 10.3390/audiolres16050122

**Published:** 2026-08-24

**Authors:** Gerard B. Remijn, Yuna Uzuhashi, Emi Hasuo, Kazuo Ueda, Yoshitaka Nakajima

**Affiliations:** 1 Faculty of Design, Department of Acoustic Design, Kyushu University, Fukuoka 815-8540, Japan; hasuo@design.kyushu-u.ac.jp (E.H.); ueda.psychoacoustics@kyudai.jp (K.U.); nakajima.yoshitaka.310@m.kyushu-u.ac.jp (Y.N.); 2 Faculty of Design, Graduate School of Design, Kyushu University, Fukuoka 815-8540, Japan; maiqiaoyuuna@gmail.com; 3 Acoustic Navigation Research Center, Doshisha University, Kyoto 610-0321, Japan; 4 Sound Corporation, Ltd., Fukuoka 813-0001, Japan

**Keywords:** word intelligibility, degraded speech, word familiarity, elderly listeners, temporal fine structure, amplitude envelope, mosaic speech

## Abstract

**Background/Objectives**: Mosaic speech was used to further investigate the auditory system’s temporal resolution needed for speech intelligibility. Mosaic speech is a form of degraded speech segmented in frequency × time blocks with no discernible temporal fine structure and with degraded amplitude envelope cues. **Methods**: We performed a listening experiment with mosaic speech consisting of 20 frequency bands segmented into 20, 40, 80, 160, and 320 ms. Younger listeners (<25 years; *n* = 20), with self-reported normal hearing and having passed a limited screening test, and elderly listeners (>65 years; *n* = 19) with hearing thresholds ranging from normal hearing to moderate hearing loss listened to Japanese low- and high-familiarity mosaic words and wrote down what they heard. The original words were included as control stimuli. **Results**: Although younger listeners had significantly higher intelligibility scores, elderly listeners could integrate and parse coarse blocks of mosaic speech of 20 ms and 40 ms with intelligibility scores of 84% or higher for high-familiarity words. For longer block durations, however, the elderly listeners’ intelligibility dropped rapidly for both high- and low-familiarity words. In both the young and the elderly listener groups, intelligibility reached the floor for block durations of 160 and 320 ms. Word familiarity strongly affected intelligibility scores. For blocks up to 80 ms, intelligibility was significantly higher for high-familiarity words than for low-familiarity words in both age groups, with a 10–30% difference. **Conclusions**: Elderly listeners (*n* = 19) with normal hearing to moderate hearing loss could maintain relatively high intelligibility for mosaic words segmented in blocks of 20 or 40 ms, provided the words had a high familiarity level.

## 1. Introduction

Word intelligibility in healthy listeners is affected by various talker- and listener-related factors such as talker familiarity [e.g., [1,2,3,4]], talker gender [5,6], the listener’s language background [e.g., [7,8]], and the listener’s age [e.g., [9,10,11]]. Linguistic characteristics such as word frequency [12,13,14] and the presence or absence of lexical context [15,16,17] also influence word understanding. Finally, intelligibility is affected by the listening environment, as shown in a wide range of studies on listening under noise [e.g., [18,19,20]] and/or reverberation [21,22,23], or in a multi-talker situation [24,25]. The combined results of studies with degraded speech have shown that, even under adverse listening conditions, speech processing is robust. Preservation of temporal envelope information in the speech signal is particularly important [e.g., [26,27]]. More than spectral distortions, temporal envelope distortions may substantially harm intelligibility, as shown in studies with temporally interrupted [28,29,30], smeared [31,32], or jittered speech [33]. Speech can remain intelligible, however, under rather coarse manipulations of temporal factors. For example, (locally) time-reversed speech is intelligible when segmented and reversed in blocks up to approximately 40–50 ms [34,35,36]. This temporal segment limit of intelligible speech dovetails well with the involvement of faster neural oscillations (~30 Hz) in the cortical construction of coherent speech streams over time [37,38,39,40,41].

Supporting evidence for the idea that speech can be intelligible when segmented in temporal blocks up to 40–50 ms comes from research with so-called mosaic speech [42,43,44,45,46]. Mosaic speech is a kind of noise-vocoded speech in which the signal is divided into frequency × time blocks. The energy distribution in each individual block is equalized, making mosaic speech a degraded speech with no identifiable temporal fine structure (TFS) and with degraded amplitude-envelope cues. Since the frequency × time-block dimensions can be independently manipulated, mosaic speech has been utilized to investigate the frequency- and time-resolving accuracy required for speech understanding. So far, studies have been performed with young, healthy listeners, using standardized speech databases in English, Mandarin Chinese, and Japanese [42,43,44]. Results showed that when segmented into 20 frequency bands, mosaic speech remains intelligible for blocks up to 40 ms, with average intelligibility percentages of 80% or higher. For blocks of 80 ms or longer, however, intelligibility rapidly drops [45,46].

In the present study, we further investigated the temporal resolution necessary for mosaic speech intelligibility. Intelligibility scores were obtained for mosaic words segmented into 20 frequency bands, with block durations of 20, 40, 80, 160, and 320 ms. The main focus is on whether elderly listeners over 65 years of age exhibit a similar pattern in intelligibility scores with increasing mosaic block duration as younger listeners. Due to age-related hearing loss (presbycusis), elderly listeners have increased difficulty hearing speech in adverse listening conditions, such as under noise [e.g., [47,48,49,50,51]] and reverberation [52,53]. Elderly listeners also tend to perform worse on temporal processing tasks that do not involve continuous speech masking [54,55], such as those using time-compressed [56,57] or interrupted speech [58]. For such tasks, not only presbycusis but also age-related slowing in cognitive functioning can affect speech understanding [59,60]. Speech without clear TFS cues is increasingly more difficult to grasp for elderly listeners, as shown in studies with jittered speech [33] or speech under noise [61]. Processing of temporal envelope cues in speech is also affected by age [62,63]. In view of this, in the present study we expect that younger listeners will be able to extract intelligible speech from mosaic speech blocks up to approximately 40 ms [42,43,44,45,46], whereas elderly listeners will already show a decline in intelligibility at this segment duration.

A second objective of this study is to investigate how word familiarity affects intelligibility under various mosaic block durations. The mosaic speech materials used so far consisted of words and sentences with everyday lexicon, often highly intelligible in their original form. In tandem with word frequency, word familiarity is another important linguistic factor that affects speech intelligibility. Speech recognition under noise and reverberation for listeners with normal hearing correlates strongly with vocabulary knowledge and working memory, with a clear advantage for high-familiarity words [64,65,66,67,68,69,70]. As for elderly listeners, some studies found a decline in intelligibility for low-familiarity words [71,72], though a potential compensatory effect of vocabulary knowledge has also been reported. Vocabulary knowledge (i.e., top-down knowledge; see Ref. [73]) tends to develop and peak at a relatively late age [74,75,76,77]. Due to mere exposure, elderly listeners accumulate lexical knowledge and linguistic skills that can help them compensate for age-related hearing difficulties in degraded listening conditions [78,79,80]. In the experiment reported here, the younger and elderly participants listened to Japanese high- and low-familiarity mosaic words, and wrote down what they heard. Our aim was to express the intelligibility difference between high- and low-familiarity words, if any, in percentages across the two age groups. This could indirectly hint at differences in the use of top-down lexical knowledge between both listener groups.

## 2. Experiment

### 2.1. Materials and Methods

#### 2.1.1. Participants

A group of young and elderly participants joined the experiment. The younger group consisted of 20 Japanese-native university students (11 males and 9 females). Their average age was 22 years (SD = 1), with a maximum of 24 years, and they joined the experiment on a voluntary basis. Although their hearing levels were not measured, all had passed a basic audiometry test consisting of a 1 kHz and 4 kHz tone presented to the left and right ear. This test, as a mandatory standard procedure in the general health check for students in Japan, uses 30 dB HL as the pass/fail line. Objectively, some participants therefore could have had mild hearing loss (i.e., between 20 and 30 dB HL), but all reported having normal hearing on a questionnaire following the experiment.

**Figure 3:**
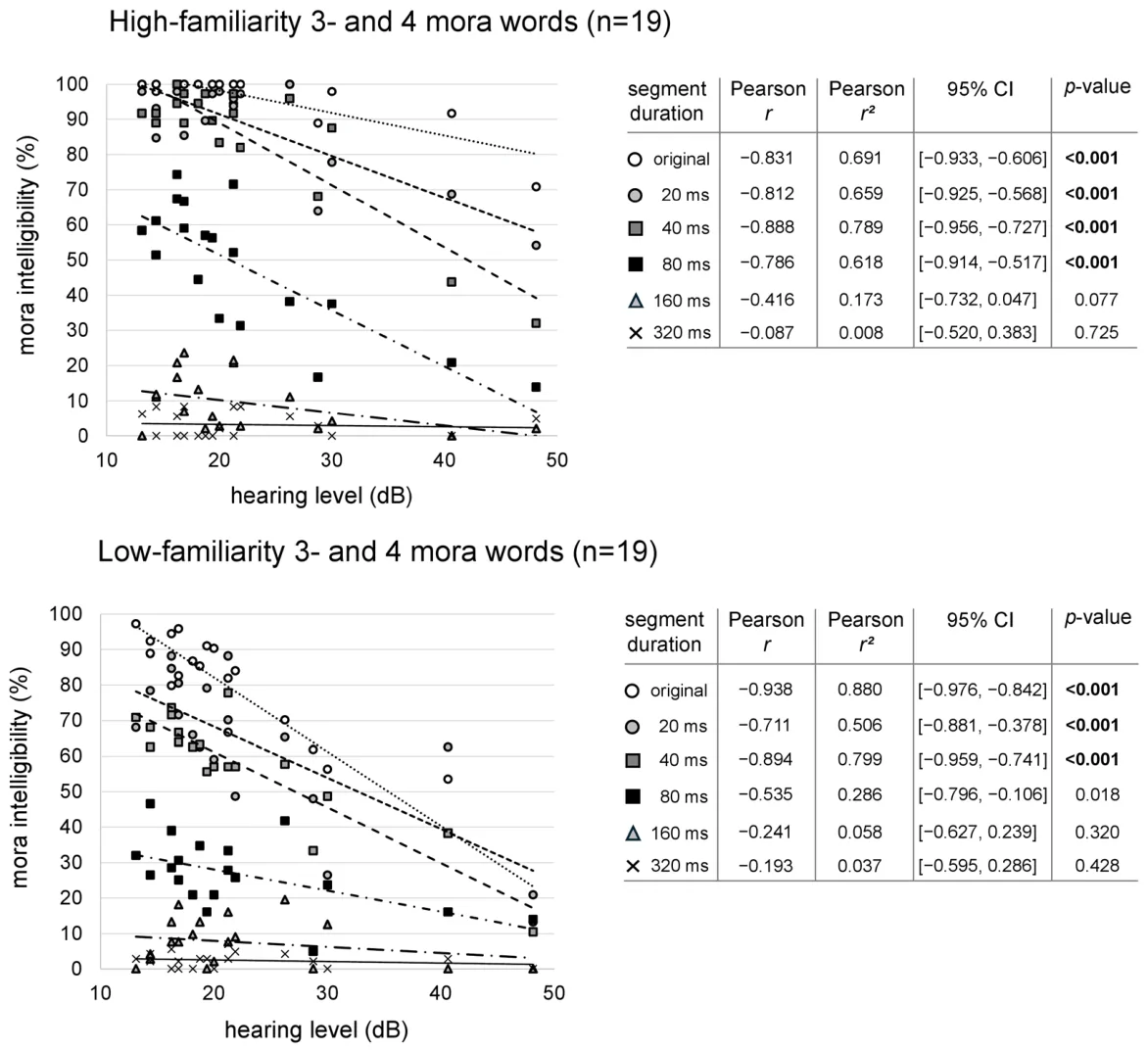
Pearson correlation coefficients between hearing level of the elderly listeners and mora intelligibility for high-familiarity words (**top**) and low-familiarity words (**bottom**). Hearing level was based on four-frequency PTA (500, 1000, 2000, 4000 Hz), averaged across both ears. Significant correlations at Bonferroni-corrected *p* < 0.004 are indicated in bold. (Original words: white circles; mosaic words with block duration of 20 ms: light gray circles; 40 ms: dark gray squares; 80 ms: black squares; 160 ms: light gray triangles; 320 ms: crosses; CI: confidence intervals).

The elderly group was 20 Japanese-native persons (10 males and 10 females) affiliated with the local “Silver Human Resources Center”. They received a book gift card for their participation. Due to technical issues, the data from one person could not be obtained correctly. The average age of the remaining 19 participants was 73 years (SD = 3), ranging from 65 to 78 years. Prior to the experiment, the hearing levels of the elderly participants (*n* = 19) were measured using an audiometer (Rion AA-K1A, Rion Co., Ltd., Tokyo, Japan). The air-conducted pure-tone hearing (threshold) levels of the elderly listeners were measured following the procedures specified by the Japan Audiology Society [81]. Measurements were performed at 250, 500, 1000, 2000, 4000, and 8000 Hz for both ears, resulting in average hearing levels of 10.26 (SD = 7.97), 15.79 (SD = 8.58), 18.95 (SD = 10.34), 25.26 (SD = 11.91), 28.95 (SD = 14.98), and 53.82 (SD = 17.57) dB HL, respectively, across both ears. Pure tone average (PTA) was calculated separately for the left ear and the right ear over 500 Hz, 1000 Hz, 2000 Hz, and 4000 Hz, and then averaged. PTA combined over both ears for the elderly was 22.2 dB HL (SD = 12.7), with a median of 19.4 dB HL, ranging from 13.1 to 48.1 dB HL. (Individual PTA values can be seen in Figure 3 in Section 3 below.) The experimental procedures were pre-approved by the local Ethics Committee (Approval No. 70-13). All participants provided written informed consent to their participation after the procedures and the task had been explained to them.

#### 2.1.2. Stimuli

1. Word selection and recording

Words were selected from the “NTT Vocabulary Database: Reiwa version of the Japanese Word Familiarity Database”, consisting of over 160,000 words [82]. Japanese has a one-to-one relation between units of language and units of writing [83]. This enables word intelligibility assessments with a high degree of accuracy. Japanese words with 3 or 4 “morae” (syllable-like units) were used [84]. Since the majority of Japanese adjectives end in the vowel /i/ and Japanese verbs end in /u/, including these would result in disproportionately high percentages correct for the word-final morae. We therefore only included Japanese nouns. Morae in Japanese nouns, however, also do not occur fully independently. Statistically, single-vowel morae naturally occur more often in Japanese, while morae like /n/ or the “small” /tsu/ cannot start a noun on their own [83].

In the database, word familiarity is indicated on a scale from 1 to 7, with a higher rating indicating a higher average familiarity to those who evaluated the words. High-familiarity words were selected among nouns with an average familiarity rating between 6 and 7. These consisted of highly or extremely familiar words encountered frequently in daily life. Low-familiarity words were selected from nouns rated on average between 1 and 2. Words given this rank were highly or completely unfamiliar to the evaluators. For the low- and high-familiarity 3-morae and 4-morae sets, 118 words were selected, constituting 472 words in total.

Speech recordings of the selected nouns were obtained from a native Japanese female and male speaker, each 22 years old. Recordings took place in a sound-attenuated booth, with a background noise level below 23 dB (A). Each speaker pronounced the 472 words three times in separately randomized sets, with a break between sets. No specific stress patterns were prescribed; speakers were instructed to use their natural Japanese intonation. If the speaker felt they had made a mistake while uttering a single word, or wished to retry, they were permitted to repeat a word multiple times. Recordings were made maintaining a 15 cm distance between the microphone (Shure KSM44A, Shure Inc., Niles, IL, USA) and the speaker’s mouth. The pronunciations were recorded (Tascam DR-100, Teac Corp., Tokyo, Japan) in .wav format, using linear PCM (Pulse Code Modulation) at a sampling frequency of 44,100 Hz with 24-bit quantization. The recorder’s gain was fixed at a level where the peak gain value was approximately 5 dB below the maximum recording level in reference to the recorder’s level meter. This allowed sufficient margin to prevent digital clipping.

Following recording, a final selection was made across the three recordings per speaker by the second author. Word recordings were selected that were free of misreadings and naturally pronounced, in which no unvoiced consonants remained unpronounced. Words whose pitch accent patterns differed from those in the “NHK Japanese Pronunciation and Accent Dictionary” [85] were excluded. As a final check, a 23-year-old Japanese-native male speaker, naïve as to the purpose of the speech files, confirmed whether the selected words were clearly pronounced. The final selection consisted of 60 words each for the four word conditions, totaling 240 words for use in the experiment.

2. Mosaic speech generation

**Figure 1:**
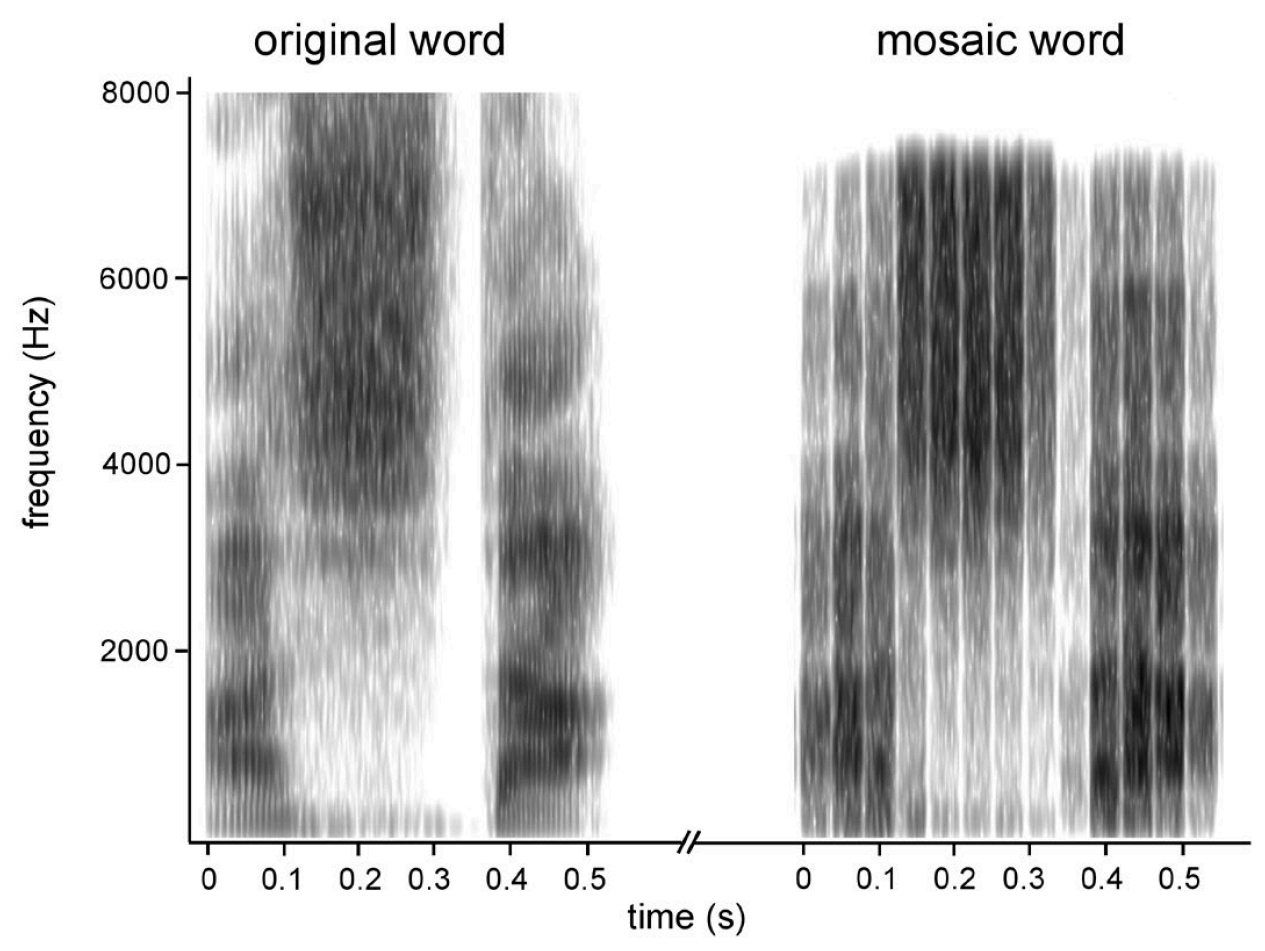
Spectrograms of example stimuli used in the experiment.

Mosaic speech stimuli were generated from the original-speech stimuli. Mosaic speech was generated as follows (see also Refs. [42,43,44,45,46]). First, the original speech was segmented into 20 bandpass filters, covering a range from 50 to 6400 Hz according to a critical-band filter bank ([86]; see also [87]). Sound energy was then calculated for speech segment durations of 20, 40, 80, 160, and 320 ms by summing the squares of the instantaneous amplitudes and subsequently averaged over these segment durations. Following this, white noise of the same duration as that of the speech signal was generated and divided into the same 20 critical-band frequency ranges, with sound energy averaged over the same five segment durations as used for the speech. For each segment, cosine-shaped rise and fall times were set to 4 ms. Subsequently, the amplitude for each band noise was adjusted to make its total sound energy equal to that of its counterpart in the processed speech signal. By connecting the segmented band-noise blocks together on the time-frequency plane, mosaic speech was generated. Speech stimuli were made using custom-made code in J language (Version J602). Figure 1 shows the spectrogram of an original word and a mosaic word. Original words were included in the stimulus set as control conditions, resulting in a pool of 2880 stimuli (240 words × 2 speakers × 6 (5 mosaic block durations + original words)).

#### 2.1.3. Equipment

The stimuli were diotically presented to participants in the sound-proof booth at a presentation level of 60 dB (A) on average. The stimuli were presented via a notebook PC (MacBook Air) located outside the booth, a digital-to-analog converter (Edirol UA-1X, Roland Corp., Hamamatsu, Japan), a low-pass filter (NF Circuit Design DV-04, NF Corp., Yokohama, Japan, cut-off 14 kHz), a graphic equalizer (Applied Research and Technology EQ335, Yorkville Sound Inc., Niagara Falls, NY, USA), and a headphone amplifier (Stax SRM-323A, Stax Ltd., Saitama, Japan) to the participant’s headphones (Stax SR-303). Sound levels were measured via a sound level meter (Aco Type 6240, ACO Co., Ltd., Tokyo, Japan) with a microphone (Brüel & Kjær, type 4134, Brüel & Kjær Sound & Vibration Measurement A/S, Nærum, Denmark), mounted on an artificial ear (Brüel & Kjær, type 4153).

#### 2.1.4. Procedure

The participant was seated in a chair in the sound-proof booth and received an explanation about the experiment and the task. They then received an explanation about the use of the experiment’s interface, which was created using PsychoPy (Version 2023.2.3). Participants listened to each word twice, with a silent interval between the words, and were asked to write down what they had heard in Japanese hiragana on a response sheet. They were instructed to write down exactly what they heard without trying to guess the content or interpret the meaning. They were told that each mora should be written down in a separate single square on the response sheet. They were also asked to indicate perceived contraction, elongation, or gemination (or a glottal stop) in the stimulus, also in a separate single square on the response sheet. For speech parts they could not hear, they were instructed to fill in the corresponding mora position on the response sheet with a period (“.”). If they could not identify any of the morae in a stimulus, they were instructed to put a checkmark (“✔”) in the checkbox next to the question number on the response sheet.

Prior to the experiment, 12 practice trials were conducted with words not used in the experiment. During the practice trials, the experimenter waited inside the booth and confirmed that the participant understood the experimental procedure and response method. During the experimental trials, the experimenter waited outside the booth. The experiment was self-paced in that the participant could press a button to start the next trial, taking breaks in between as they pleased. The stimuli were randomly presented with balanced conditions, such that the number of morae in the words (2 conditions: 3 or 4 morae), the gender of the speaker (2 genders: male or female), word familiarity (2 conditions: high- and low-familiarity words), the number of words for each familiarity level (3 words), and the block duration of the mosaic words (5 durations of 20, 40, 80, 160, and 320 ms, and the original-word condition as control) were the same for each participant. Each participant thus received (2 × 2 × 2 × 3 × 6 =) 144 stimuli in total, each made from a different word selected from the 240 words. Though each participant received a different word selection, the same word could be used for different participants, yet for a different (original or mosaic) word condition. The maximum word overlap between two participants was 120 words out of 144 stimuli, while the minimum was 48 words. The stimuli were presented in 3 sessions of 48 trials each. An optional rest period was provided between each session. The total duration of the experiment was approximately 55 min for both listener groups.

## 3. Results

**Figure 2:**
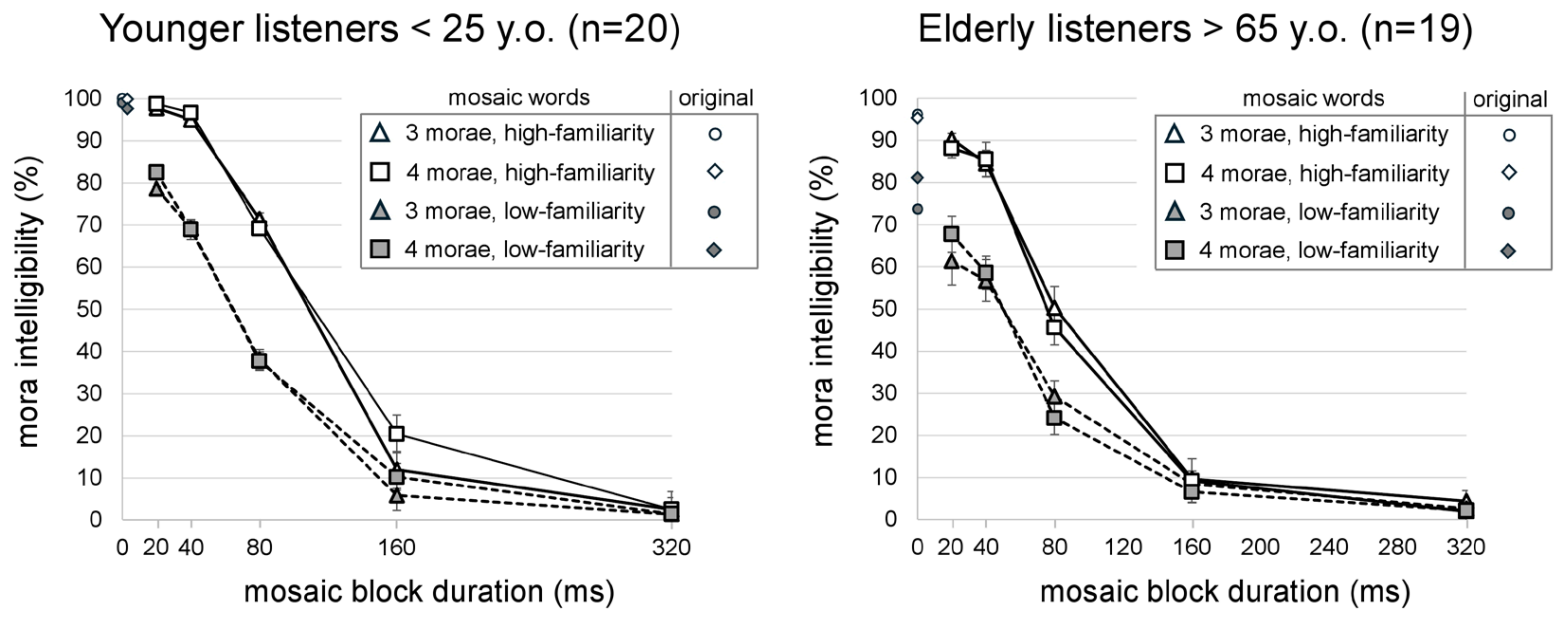
Mora intelligibility as a function of mosaic block duration for younger (**left**) and elderly listeners (**right**). Intelligibility scores for high-familiarity original and mosaic words are indicated by white symbols, while those for low-familiarity original and mosaic words are indicated by gray symbols.

Word intelligibility was calculated by assigning one point per correct mora and converting accumulated scores into percentages correct. Figure 2 shows the intelligibility percentages for the young listeners (<25 years of age; *n* = 20) and the elderly listeners (>65 years of age; *n* = 19). For both the 3- and 4-mora words, a very similar pattern of results was found. For younger listeners, the intelligibility of high-familiarity words was 95% or higher for original words and mosaic words segmented into 20 and 40 ms, but dropped to about 70% for the 80 ms block duration. Intelligibility reached the floor for words with 160 ms and 320 ms blocks. Low-familiarity word intelligibility was above 95% only in the original-word condition. For the 20- and 40-ms block durations, intelligibility declined to about 80% and just below 70%, respectively. Intelligibility for 80 ms low-familiarity words dropped below 40%—just over 30% lower than for 80 ms high-familiarity words. For words segmented in 160 ms and 320 ms, intelligibility differences between high- and low-familiarity words were small due to the floor effect.

For elderly listeners, high-familiarity words were very intelligible for original words and mosaic words with block durations of 20 and 40 ms as well (84% or over). Intelligibility decreased rapidly to about 45% for words with 80 ms blocks, and to below 10% for words with the longest block durations. Low-familiarity original words were not fully intelligible for elderly listeners, with a percentage of 74% for 3-morae words and 82% for 4-morae words. For mosaic words segmented into 20 or 40 ms blocks, intelligibility was roughly between 55 and 70%, but moved below 30% for blocks of 80 ms. For the longest block durations of 160 ms and 320 ms, intelligibility of low-familiarity words also neared the floor. Compared with younger listeners, intelligibility scores for both high- and low-familiarity words were lower for elderly listeners, but differences over 20% were only found for low-familiarity, 3-mora original words and high-familiarity, 3- and 4-mora mosaic words with 80 ms blocks.

Figure 3 shows the Pearson correlation coefficients between the individual hearing levels of the elderly listeners (*n* = 19), based on the four-frequency PTA averaged across both ears, and their intelligibility scores for original words and mosaic words. Significant negative correlations at the Bonferroni-corrected *p*-value of 0.05/12 = 0.004 were found for high-familiarity original words and mosaic words up to 80 ms blocks. Significant negative correlations between hearing level and intelligibility were found for low-familiarity original words and mosaic words up to 40 ms blocks. Overall, a decrease in word intelligibility was correlated with an increase in the elderly’s hearing loss. Since audiometry was not performed, correlation coefficients for young listeners were not obtained.

The data for the mosaic word conditions were analyzed in a series of Generalized Linear Mixed-Effects Models (GLMMs) with a beta-binomial distribution and a logit link function. The data set can be obtained here: https://doi.org/10.6084/m9.figshare.32869541 (accessed on 2 July 2026). We proceeded from the idea that the intelligibility of each mora in a word was correlated with that of the other morae in the word. Morae in low-familiarity words would all have lower intelligibility, while the morae in a high-familiarity word were all assumed to have a higher intelligibility. Instead of a standard binomial GLMM, in which each mora in a word would have a fixed intelligibility probability, we opted for a beta-binomial GLMM. The beta-binomial model assumes that each word gets its own intelligibility probability and all morae belonging to the same word share that word’s specific probability. The GLMM code and output are available in the Supplementary Materials.

The original word data were not included in the GLMM, but please see the Supplementary Materials for the GLMM results for the full data set, including the original word data and direct comparisons between the original word condition and the mosaic speech conditions. The Supplementary Materials also include the results of a GLMM with PTA as a continuous predictor, performed over the elderly listeners’ data. Both GLMMs confirm the model’s results described below. The GLMM over the elderly listeners’ data aligns with the correlation results, in that intelligibility decreased significantly as hearing loss increased for mosaic blocks up to 160 ms for high-familiarity words and up to 80 ms for low-familiarity words. GLMMs were run with custom-made code in R (version R 4.5.2) over the number of correct and the number of missed morae (“successes and failures”) in each 3- or 4-morae word.

For the main analysis, several nested models were compared via Likelihood Ratio Tests. The analyses showed that the optimal fit was achieved by a full model with *block duration*, *word familiarity*, and *age* as fixed effects, sum-coded as categorical predictors, along with their two- and three-way interactions. *Morae number* and *trial number* were included as mean-centered continuous covariates, with the former to control for word length, and the latter to control for possible learning or fatigue effects as the experiment progressed. Trial number indicated the order of presentation of the 144 trials for each participant. Interactions between trial number and, for example, age were omitted to prevent model overparameterization and to preserve statistical power.

*Participant ID* and *word ID* were included as random intercepts to account for repeated measures. Word ID was a number between 1 and 240, representing a high- or low-familiarity word stimulus. We also included a participant-specific random slope for morae number to see whether the effect of word length varied across participants. Random slopes for categorical experimental conditions, such as for block duration, were omitted to prevent model singularity and ensure parameter convergence. Model comparison showed that adding word ID and trial number to an initial model with participant ID as a random effect significantly improved the model’s fit. Adding trial number significantly reduced the AIC (Akaike Information Criterion) by 19.9 points. The intelligibility difference between words in the stimulus set was a major source of variance, significantly reducing the AIC by 102.9 points, resulting in an AIC of 7755.9 points for the best-fit model.

The results of the best-fit model were as follows. Type III Wald chi-square analysis of deviance (Table 1) revealed significant main effects of block duration [$\chi^{2}\left(4\right)=2496.01,\;p<0.001$], word familiarity [$\chi^{2}\left(1\right)=140.55,p<0.001$], age [$\chi^{2}\left(1\right)=9.20,p=0.0024$], and trial number [$\chi^{2}\left(1\right)=14.87,p<0.001$]. Morae number was not significant [$\chi^{2}\left(1\right)=0.24,p=0.624$]. Significant interactions existed between block duration × word familiarity [$\chi^{2}\left(4\right)=137.50,p<0.001$], block duration × age [$\chi^{2}\left(4\right)=27.40,p<0.001$], and word familiarity × age [$\chi^{2}\left(1\right)=14.36,p<0.001$]. The three-way interaction was not statistically significant [$\chi^{2}\left(4\right)=1.46,p=0.835$]. Post hoc comparisons (Tukey-adjusted), as shown in Supplementary Materials, showed that younger listeners achieved significantly higher mora intelligibility than elderly listeners for high-familiarity words (OR=2.61,p<0.001). In contrast, for low-familiarity words, intelligibility did not differ significantly between younger and elderly listeners (OR=1.40,p=0.178).

As for block duration, post hoc pairwise comparisons (Tukey-adjusted) revealed that intelligibility decreased significantly across every adjacent duration increase (*p* < 0.05). Within high-familiarity words, relatively large intelligibility drops could be observed between 40 ms vs. 80 ms (Odds Ratio (OR)=11.02,p<0.0001) and 80 ms vs. 160 ms (OR = 14.61, p<0.0001). Low-familiarity word intelligibility also degraded steadily across the 40 ms vs. 80 ms (OR=4.00,p<0.0001) and 80 ms vs. 160 ms (OR=9.51,p<0.0001) block duration expansions. As for trial number, the significant effect (with β=0.003) indicates that intelligibility slightly increased over the course of the experiment.

Table 2 shows that baseline intelligibility varied substantially across words ($\sigma^{2}=0.30,\;\mathrm{SD}=0.55$) and participants ($\sigma^{2}=0.36,\;\mathrm{SD}=0.60$). Some words were naturally less intelligible than others, and some participants performed better than others, regardless of the stimulus conditions. Although individual sensitivity to morae number varied slightly ($\sigma^{2}=0.09,\;\mathrm{SD}=0.29$), this effect did not correlate with overall intelligibility (r=−0.01).

## 4. Discussion

The results showed near-perfect intelligibility for high-familiarity words in younger listeners for mosaic blocks of 20 and 40 ms, and close to 70% even for 80 ms blocks. This pattern of results corroborates earlier findings with mosaic speech with younger listeners [42,43,44,45,46]. For elderly listeners over 65 years of age, intelligibility scores of 84% or higher were found for mosaic speech segmented up to 40 ms for high-familiarity words. For 80 ms blocks, however, intelligibility dropped below 50%—about 30% lower than that for younger listeners. As the first objective of this study, this confirms that elderly listeners with normal hearing to moderate hearing loss could parse and integrate coarse blocks (up to 40 ms) of mosaic speech relatively well, provided the stimuli were high-familiarity words.

Although neurophysiological data on mosaic speech have yet to be obtained, the present behavioral data are consistent with the dual-time window theory of speech processing [37,38,39,40,41]. According to this theory, our auditory brain uses two temporal scales of neural oscillatory activity to construct coherent speech streams over time. One is a slow oscillation of about 4–8 Hz (segments of 125–250 ms), said to work on the speech syllable level. The other is a fast oscillation of about 30 Hz (segments of ~33 ms), said to work on the speech phoneme level. Earlier findings using mosaic speech and other degraded speech forms have suggested a correspondence between the 40 ms segment limit for intelligible speech and the fast oscillation frequency involved in extracting phonetic information (see also Refs. [42,43]). The present data imply that even for listeners over 65 years of age, phoneme-level blocks of degraded speech material can be quite intelligible. The potential correspondence with fast neural oscillations in the aging brain is a topic for future research.

For mosaic speech segmented in 80 ms blocks or longer, intelligibility was around 50% or below for elderly listeners, for both high- and low-familiarity words. Mosaic speech does not have a clearly identifiable TFS, and taking advantage of TFS in speech becomes more challenging as we grow older [88], even without any serious hearing problems [60]. Without TFS, speech perception is strongly affected in older listeners, as shown in studies with jittered speech [33] or speech under noise [61]. The absence of smooth envelope transitions in mosaic speech would also make processing difficult for elderly listeners in particular. Both human behavioral data and animal models demonstrate that age-related changes in the processing of envelope cues could be a prominent cause of reduced speech intelligibility [62,63]. Our present results showed that an increase in the elderly listener’s hearing levels strongly correlated with intelligibility decline for mosaic speech segmented up to 80 ms for high-familiarity words and up to 40 ms for low-familiarity words (Figure 3). However, while effects of age on speech intelligibility have been extensively reported so far (e.g., Refs. [58,59,89,90,91]), correlations do not express causality, and we cannot attribute the intelligibility differences strictly to hearing thresholds, because our design does not separate peripheral auditory loss, central hearing deficits, and cognitive aging. This limitation is further discussed below.

Regarding the second objective of this study, we expected that high-familiarity mosaic words would be more intelligible when degraded than low-familiarity mosaic words, given the familiarity effects reported so far in speech studies [64,65,66,67,68,69,70]. Our goal was to express intelligibility differences in percentages across mosaic block durations and age groups. Intelligibility was indeed significantly higher for high-familiarity words than for low-familiarity words in conditions without a ceiling or floor effect. For mosaic speech with 20, 40, and 80 ms blocks, the difference was quite substantial: roughly 10–30% in both listener groups. A common framework describing the role of top-down vocabulary knowledge on intelligibility is the Ease of Language Understanding (ELU) model [48,92,93]. In this model, the phonological representation of incoming speech is matched against lexical representations in the listener’s long-term memory. If correspondence is found, word understanding is automatic and does not require further top-down processing. To achieve this, however, the incoming speech signal should be of sufficient quality to make a phonological match with the listener’s stored knowledge. If such phonological cues are degraded and a match is not immediately found, the listeners must use cognitive resources in a top-down way to interpret the speech. Cochlear implant users, for example, can make good use of cognitive-linguistic top-down knowledge provided the bottom-up input is of sufficient quality [94]. In the present experiment, however, even low-familiarity words presented in their original, undegraded form seemed less intelligible for elderly listeners than for younger listeners (24% and 16% difference for 3- and 4-morae words). Bottom-up cues in the acoustic signal were clearly present in these words, and although these may not have triggered a lexical match in the listener’s stored vocabulary, the morae could have been correctly written down according to phonological content. In Japanese, there is a strong sound-to-mora correspondence, i.e., a shallow orthography, yet the elderly listeners had difficulty with the low-familiarity words. Within the ELU model’s framework [48,92,93], the present results show that even for relatively short 20 ms to 40 ms blocks of (degraded) mosaic speech, intelligibility decreases when listeners need to rely solely on bottom-up phonological cues, as when confronted with low-familiarity words that do not automatically trigger a match with stored lexical knowledge.

In relation to this, from the results we cannot say that top-down vocabulary knowledge assisted mosaic speech perception more in elderly listeners than in younger listeners. No significant intelligibility difference was found between both age groups for low-familiarity mosaic words. While some studies have shown intelligibility gains in normal-hearing elderly due to their extended vocabulary or attentional resources (e.g., [78,95]), the present results seem to suggest that distortions (or absence) of TFS and amplitude envelope cues cannot easily be compensated for with vocabulary knowledge in the elderly. It needs to be said, though, that the stimuli were selected among words with the highest or the lowest familiarity ratings in the database, i.e., from words either used in everyday conversation or from those rarely encountered in life. Furthermore, the words were not only degraded but also presented in isolation. Even though the words were presented twice, and repetition of speech can improve intelligibility in degraded listening situations [96], the participants could not make use of top-down linguistic context known to improve intelligibility [17,97,98]. The profound intelligibility difference of 10–30% between low- and high-familiarity words for mosaic blocks up to 80 ms thus could be close to the maximum that can be induced with existing words. Finally, it is also important to note that the analyses were done over morae intelligibility, not full-word intelligibility. Full-word intelligibility was very limited for low-familiarity words, especially in elderly listeners: the combined intelligibility percentages over 3- and 4-morae words for both speakers showed that original words reached an intelligibility score of 52%, while that for the shortest mosaic block duration of 20 ms dropped already to 26%. Intelligibility for low-familiarity mosaic words with 40 ms and 80 ms blocks already reached the floor (17% and 1%, respectively).

## 5. Conclusions

In conclusion, the present study adds to our understanding of the perception of degraded high- and low-familiarity words in young and elderly listeners. The results show that even as we age, our auditory system is able to integrate and parse rather coarse segments of degraded speech information. Strings of segments up to 40 ms without clear TFS and amplitude envelope cues can be quite intelligible, provided hearing loss is limited and familiar speech is used. Presuming that intelligibility over 80% for mosaic blocks up to 40 ms can be considered relatively high for degraded speech, this would correspond to a processing window at the phoneme-duration level.

Several limitations should be mentioned. First, the high- and low-familiarity words selected from the database were not strictly matched in terms of factors such as phonotactic probability, neighborhood density, accent pattern, and mora composition. Japanese has a consonant-vowel mora structure [83] and four main accent patterns [99]. Statistically, these factors should be comparable between the high- and low-familiarity word sets, but we cannot rule out that they contributed to the difference in intelligibility scores. A more controlled word selection should remedy this in future research. Next, while the elderly participants received audiometry testing, the younger participants did not. We assume they had normal hearing based on their self-reporting and the fact that they had passed the standard audiometry test as part of the mandatory health check. However, they technically could have had mild hearing loss.

Another methodological limitation is that we did not perform any explicit word vocabulary testing or cognitive testing in this study—the elderly participants joined on a voluntary basis and were preselected by the elderly organization that cooperated. The age-group difference for high-familiarity mosaic words could be due to differences in peripheral hearing abilities as well as central (cognitive) processing, but the present study cannot draw any clear conclusions on this. To better understand the current results, in future studies a Japanese vocabulary test or a kanji aptitude test measuring the listener’s knowledge of Chinese characters used in Japanese writing should be administered. Cognitive testing could be undertaken, for example, by using the Japanese version of the Mini-Mental State Exam for elderly persons [100]. Finally, another factor that may have contributed to the results is that the younger group in our study were university students, mostly trained as acoustic engineers, who were used to performing listening experiments. The elderly listeners had less experience participating in experimental research, which may have contributed to the difference with the younger group. The significant effect of trial number might indicate that intelligibility slightly increased as the experiment progressed, but insight into learning effects or listener fatigue [101,102] requires a more targeted investigation than the present study.

## Figures and Tables

**Table 1:** Generalized linear mixed model results: fixed effects significance.

Fixed Effects Significance Predictor	(Type III)	*df*	*p-Value*
	χ^2^		
**(Intercept)**	**25.03**	**1**	**<0.001**
**Block duration**	**2496.01**	**4**	**<0.001**
**Word familiarity**	**140.55**	**1**	**<0.001**
**Age**	**9.20**	**1**	**0.002**
Morae number (cent.)	0.24	1	0.624
**Trial number (cent.)**	**14.87**	**1**	**<0.001**
**Block duration × Word Familiarity**	**137.50**	**4**	**<0.001**
**Block duration × Age**	**27.40**	**4**	**<0.001**
**Word Familiarity × Age**	**14.36**	**1**	**<0.001**
Block duration × Word Familiarity × Age	1.46	4	0.835

χ^2^ = Type III Wald chi square; df = degrees of freedom; cent. = centered. Significant effects are indicated in bold.

**Table 2:** Generalized Linear Mixed Model results: Random Effects Variance.

Random Effects Variance Groups	Effect	Variance	Standard Deviation
Word ID	random intercept	0.304	0.552
Participant ID	random intercept	0.357	0.598
Morae number	random slope	0.085	0.292

## Data Availability

The GLMM code in R and the output are available as Supplementary Material. The data set can be obtained here: https://doi.org/10.6084/m9.figshare.32869541.

## Supplementary Materials

The following supporting information can be downloaded at: https://www.mdpi.com/article/10.3390/audiolres16050122/s1, Supplementary Material S1: R script of GLMM and output report; Supplementary Material S2: Results of the GLMM analysis as under S1, with the full data set including the original-word data; Supplementary Material S3: GLMM with the intelligibility data from the elderly participants and four-frequency PTA combined across ears as a continuous predictor.

## Abbreviations

The following abbreviations are used in this manuscript:

ms	millisecond
TFS	temporal fine structure
SD	standard deviation
PTA	pure-tone average
HL	hearing level
Hz	Hertz
dB	decibel
PCM	pulse-code modulation
NTT	Nippon Telegraph and Telephone Corporation
NHK	Nippon Hōsō Kyōkai (Japan Broadcasting Corporation)
GLMM	generalized linear mixed model
ID	identifier
AIC	Akaike Information Criterion
df	degrees of freedom
ELU	ease of language understanding
