# Supplementary material for: The Temporal Resolution Needed for Speech Intelligibility Assessed with Mosaic Speech: Effects of Block Duration, Age, and Word Familiarity
Source: Audiol Res. 2026 Aug 24;16(5):122. doi: 10.3390/audiolres16050122 (PMC13600226; doi:10.3390/audiolres16050122)
Supplement: Supplementary file 1 [file audiolres-16-00122-s001.zip › audiolres-4431308-supplementary.pdf]

Supplementary Material S1

R script of GLMM and Output report

Note: R code smoothed using AI. Output report generated with AI support.

```
# ================================================================================
# 1. GLOBAL SETTINGS & LOAD LIBRARIES
# ================================================================================
# Global option: Set orthogonal contrasts BEFORE data or factors are processed
options(contrasts = c("contr.sum", "contr.poly"))

library(glmmTMB)   # For Beta-Binomial GLMM modeling
library(car)       # For Anova() Type III Wald tests
library(emmeans)   # For post-hoc pairwise comparisons
library(ggplot2)   # For generating publication figures
library(DHARMa)    # For GLMM residual diagnostics

# ================================================================================
# 2. DATA PREPARATION & FACTOR CODING
# ================================================================================
# Load cleaned dataset
data_Ex1 <- read.csv("Remijn et al data cleaned.csv", check.names = FALSE)

# Compute failed moras for the binomial/beta-binomial matrix format
data_Ex1$Moraefail <- data_Ex1$Morae_number - data_Ex1$Moraecorrect

# Ensure categorical factors are formatted with explicit sum contrasts for Type III ANOVA
fixed_factors <- c("Block duration", "Word Familiarity", "Age")
random_factors <- c("Participant ID", "Word ID")

# Convert to factor
for (f in c(fixed_factors, random_factors)) {
  data_Ex1[[f]] <- as.factor(data_Ex1[[f]])
}

# Explicitly assign contr.sum to fixed categorical predictors
for (f in fixed_factors) {
  contrasts(data_Ex1[[f]]) <- contr.sum
}

# Convert numeric predictors safely and check for NAs
data_Ex1$`Trial ID` <- as.numeric(as.character(data_Ex1$`Trial ID`))
data_Ex1$Morae_number <- as.numeric(as.character(data_Ex1$Morae_number))

if (any(is.na(data_Ex1$`Trial ID`)) || any(is.na(data_Ex1$Morae_number))) {
    warning("NAs detected during numeric conversion of Trial ID or Morae_number. Please check source data.")
}
```

```
# Mean-center continuous variables (as flat vectors to prevent matrix structure issues)
data_Ex1$Trial_c <- as.vector(scale(data_Ex1$`Trial ID`, scale = FALSE))
data_Ex1$Morae_number_c <- as.vector(scale(data_Ex1$Morae_number, scale = FALSE))

# Verify experimental cell balance
print("--- Check Factor Levels and Counts ---")
print(table(data_Ex1$`Block duration`, data_Ex1$`Word Familiarity`))

# ==============================================================================
# 3. DEFINE AND COMPARE NESTED BETA-BINOMIAL MODELS
# ==============================================================================
# Model 1: BASELINE MODEL
model_base <- glmmTMB(
  cbind(Moraecorrect, Moraefail) ~ `Block duration` * `Word Familiarity` * Age +
    Morae_number_c +
    (1 + Morae_number_c | `Participant ID`),
  data = data_Ex1,
  family = betabinomial(link = "logit")
)

# Model 2: ADDS CONTINUOUS TRIAL (Task progression / fatigue control)
model_with_trial <- glmmTMB(
  cbind(Moraecorrect, Moraefail) ~ `Block duration` * `Word Familiarity` * Age +
    Morae_number_c +
    Trial_c +
    (1 + Morae_number_c | `Participant ID`),
  data = data_Ex1,
  family = betabinomial(link = "logit")
)

# Model 3: FULL MODEL (Adds item-level random intercept for Word ID)
model_full <- glmmTMB(
  cbind(Moraecorrect, Moraefail) ~ `Block duration` * `Word Familiarity` * Age +
    Morae_number_c +
    Trial_c +
    (1 + Morae_number_c | `Participant ID`) +
    (1 | `Word ID`),
  data = data_Ex1,
  family = betabinomial(link = "logit")
)

# Sequential Likelihood Ratio Tests
print("--- Model Comparisons (AIC / LRT) ---")
model_comparison <- anova(model_base, model_with_trial, model_full)
print(model_comparison)
write.csv(as.data.frame(model_comparison), "model_comparison_table.csv", row.names = TRUE)

# ==============================================================================
# 4. STATISTICAL INFERENCE, VARIANCE CHECK & RESIDUAL DIAGNOSTICS
# ==============================================================================
```

```
# Summary of the full model
summary(model_full)

# --- RANDOM SLOPE VARIANCE CHECK ---
vc <- VarCorr(model_full)
participant_var <- vc$cond$`Participant ID`
slope_var <- participant_var["Morae_number_c", "Morae_number_c"]

if (is.na(slope_var) || slope_var < 0.001) {
  warning(
    paste0(
      "\n---------------------------------------------------------------------\n",
      "WARNING: The random slope variance for 'Morae_number_c' across\n",
      "participants is near zero (Variance = ", round(slope_var, 6), ").\n",
      "This indicates potential model overparameterization or singular fit.\n",
      "Consider simplifying the random effect to '(1 | `Participant ID`)'.\n",
      "---------------------------------------------------------------------\n"
    )
  )
} else {

  cat(paste0("\n[OK] Random slope variance for 'Morae_number_c' across participants is ", round(slope_var,
4), ".\n\n"))
}

# Type III Wald Chi-Square Tests
anova_results <- Anova(model_full, type = "III")
print("--- Type III Wald Chi-Square Tests ---")
print(anova_results)

# DHARMa Residual Diagnostics for Beta-Binomial
simulationOutput <- simulateResiduals(fittedModel = model_full, plot = TRUE)
testDispersion(simulationOutput)

# ==============================================================================
# 5. POST-HOC EMMEANS COMPARISONS & INTERACTION TABLES
# ==============================================================================
# 5A. Pairwise Comparisons for Block duration Sliced BY Word Familiarity
emm_block_fam <- emmeans(
  model_full,
  ~ `Block duration` | `Word Familiarity`,
  data = data_Ex1,
  type = "response"
)
pairs_block_fam <- pairs(emm_block_fam, adjust = "tukey")

print("--- Pairwise Comparisons of Block duration WITHIN Word Familiarity (Tukey Adjusted) ---")
print(pairs_block_fam)

# 5B. Pairwise Comparisons for Block duration Sliced BY Age
```

```
emm_block_age <- emmeans(
  model_full,
  ~ `Block duration` | Age,
  data = data_Ex1,
  type = "response"
)
pairs_block_age <- pairs(emm_block_age, adjust = "tukey")

print("--- Pairwise Comparisons of Block duration WITHIN Age Groups (Tukey Adjusted) ---")
print(pairs_block_age)

# ---------------------------------------------------------------------------
# 5D. FULL INTERACTION TABLES (2-WAY & 3-WAY INTERACTION MEANS & PAIRWISE)
# ---------------------------------------------------------------------------

# --- 2-WAY INTERACTION: Block duration x Word Familiarity ---
emm_2way_block_fam <- emmeans(
  model_full,
  ~ `Block duration` * `Word Familiarity`,
  data = data_Ex1,
  type = "response"
)
pairs_2way_block_fam <- pairs(emm_2way_block_fam, adjust = "tukey")

# --- 2-WAY INTERACTION: Block duration x Age ---
emm_2way_block_age <- emmeans(
  model_full,
  ~ `Block duration` * Age,
  data = data_Ex1,
  type = "response"
)
pairs_2way_block_age <- pairs(emm_2way_block_age, adjust = "tukey")

# --- 2-WAY INTERACTION: Word Familiarity x Age ---
emm_2way_fam_age <- emmeans(
  model_full,
  ~ `Word Familiarity` * Age,
  data = data_Ex1,
  type = "response"
)
pairs_2way_fam_age <- pairs(emm_2way_fam_age, adjust = "tukey")

# --- 3-WAY INTERACTION: Block duration x Word Familiarity x Age ---
emm_3way_full <- emmeans(
  model_full,
  ~ `Block duration` * `Word Familiarity` * Age,
  data = data_Ex1,
  type = "response"
)
# Sliced comparisons across Block duration for each Age x Familiarity cell
```

```
pairs_3way_sliced <- pairs(
  emmeans(
    model_full,
    ~ `Block duration` | `Word Familiarity` * Age,
    data = data_Ex1,
    type = "response"
  ),
  adjust = "tukey"
)

# Print 3-Way Summary to Console
print("--- Estimated Marginal Means for 3-Way Interaction ---")
print(emm_3way_full)

# Save All Sliced & Interaction Tables to CSV Files

write.csv(as.data.frame(pairs_block_fam), "pairwise_block_by_familiarity_tukey.csv", row.names = FALSE)

write.csv(as.data.frame(pairs_block_age), "pairwise_block_by_age_tukey.csv", row.names = FALSE)



# Export Interaction EMMs (Probability Scale)

write.csv(as.data.frame(emm_2way_block_fam), "emm_2way_block_x_familiarity.csv", row.names = FALSE)

write.csv(as.data.frame(emm_2way_block_age), "emm_2way_block_x_age.csv", row.names = FALSE)

write.csv(as.data.frame(emm_2way_fam_age), "emm_2way_familiarity_x_age.csv", row.names = FALSE)

write.csv(as.data.frame(emm_3way_full), "emm_3way_block_x_familiarity_x_age.csv", row.names = FALSE)



# Export Interaction Pairwise Contrasts (Odds Ratios / Tukey Adjusted)
write.csv(as.data.frame(pairs_2way_block_fam), "pairwise_2way_block_x_familiarity.csv", row.names =
FALSE)

write.csv(as.data.frame(pairs_2way_block_age), "pairwise_2way_block_x_age.csv", row.names = FALSE)

write.csv(as.data.frame(pairs_2way_fam_age), "pairwise_2way_familiarity_x_age.csv", row.names = FALSE)
write.csv(as.data.frame(pairs_3way_sliced), "pairwise_3way_block_by_familiarity_and_age.csv", row.names
= FALSE)
```

Statistical Analysis Report: Beta-Binomial GLMM

EXECUTIVE SUMMARY

Metric / Area	
**Model Selection**	Model 3 (Full Model with Word ID random intercepts and continuous Trial progression) demonstrated best fit (AIC = 7755.9, LogLik = -3851.0, $p < 0.001$ vs nested baselines).
**Primary Predictors**	Block duration (Chisq = 2496.01, $p < .0001$) and Word Familiarity (Chisq = 140.55, $p < .0001$) are the strongest predictors of mora intelligibility.
**Interaction Effects**	Significant two-way interactions were observed for Block Duration × Word Familiarity ($p < .0001$), Block Duration × Age ($p < .0001$), and Word Familiarity × Age ($p < .0001$).
**Three-Way Interaction**	The three-way Block Duration × Word Familiarity × Age interaction was NOT statistically significant (Chisq = 1.46, $p = 0.835$).
**Model Validity**	DHARMa residual diagnostics confirm no overdispersion (Dispersion = 1.0036, $p = 0.768$). Random slope variance for Morae Number is robust (Var = 0.0850).

MODEL FIT & OVERALL PARAMETERS

**Model Formula**	cbind(Moraecorrect, Moraefail) ~ Block duration * Word Familiarity * Age + Morae_number_c + Trial_c + (1 + Morae_number_c | Participant ID) + (1 | Word ID)
**Distribution Family**	Beta-Binomial (Link: Logit)
**Akaike Information Criterion (AIC)**	7755.9
**Bayesian Information Criterion (BIC)**	7930.1
**Log-Likelihood**	-3851.0
**Deviance / Residual Df**	7701.9 / 4653
**Dispersion Parameter (phi)**	3.77
**DHARMa Dispersion Test Ratio (p-val)**	1.0036 ($p = 0.768$)

**Total Observations / Participants / Words**	4,680 obs / 39 participants / 240 words

Type III Wald Chi-Square Tests (model_full)

Hypothesis testing for main effects and interaction terms under orthogonal sum contrasts

Effect / Predictor	Chisq	Df	Pr(>Chisq)	Significance
**(Intercept)**	25.0328	1	5.64E-07	***
**Block duration**	2,496.0147	4	2.20E-16	***
**Word Familiarity**	140.5476	1	2.20E-16	***
**Age**	9.2030	1	0.0024	**
**Morae_number_c**	0.2406	1	0.6238	**ns**
**Trial_c**	14.8669	1	1.15E-04	***
**Block duration : Word Familiarity**	137.5000	4	2.20E-16	***
**Block duration : Age**	27.3981	4	1.65E-05	***
**Word Familiarity : Age**	14.3551	1	1.51E-04	***
**Block duration : Word Familiarity : Age**	1.4555	4	0.8345	**ns**

Fixed Effects Parameter Estimates (Conditional Model)

Logit scale coefficients, standard errors, z-values, and p-values (Sum-coded categorical variables)

Parameter Name	Estimate (Logit)	Std. Error	z value	Pr(>|z|)	Odds Ratio	Signif
(Intercept)	-0.5513	0.1102	-5.00	5.64E-07	0.5762	***
Block duration 1 (20ms)	2.8329	0.0886	31.97	2.00E-16	16.9937	***
Block duration 2 (40ms)	2.2545	0.0721	31.25	2.00E-16	9.5306	***
Block duration 3 (80ms)	0.3613	0.0563	6.41	1.41E-10	1.4351	***
Block duration 4 (160ms)	-1.8907	0.0719	-26.30	2.00E-16	0.1509	***
Word Familiarity 1 (High)	0.6460	0.0545	11.86	2.00E-16	1.9079	***
Age 1 (Group 1)	0.3259	0.1074	3.03	0.0024	1.3853	**
Morae_number_c	0.0507	0.1034	0.49	0.6238	1.0520	ns
Trial_c	0.0032	0.0008	3.86	1.15E-04	1.0032	***
Block duration 1 : Word Familiarity 1	0.6000	0.0859	6.99	2.80E-12	1.8221	***
Block duration 2 : Word Familiarity 1	0.5101	0.0700	7.29	3.15E-13	1.6654	***
Block duration 3 : Word Familiarity 1	0.0036	0.0563	0.06	0.9492	1.0036	ns
Block duration 4 : Word Familiarity 1	-0.4259	0.0701	-6.08	1.21E-09	0.6532	***
Block duration 1 : Age 1	0.3205	0.0859	3.73	1.89E-04	1.3778	***
Block duration 2 : Age 1	0.1165	0.0698	1.67	0.0952	1.1235	.
Block duration 3 : Age 1	0.1237	0.0562	2.20	0.0279	1.1317	*
Block duration 4 : Age 1	-0.1073	0.0699	-1.53	0.1251	0.8983	ns
Word Familiarity 1 : Age 1	0.1557	0.0411	3.79	1.51E-04	1.1685	***
Block duration 1 : Word Familiarity 1 : Age 1	0.0869	0.0853	1.02	0.3080	1.0908	ns
Block duration 2 : Word Familiarity 1 : Age 1	0.0293	0.0694	0.42	0.6731	1.0297	ns

**Block duration 3 : Word Familiarity 1 : Age 1**	-0.0071	0.0562	-0.13	0.9000	0.9930	**ns**
**Block duration 4 : Word Familiarity 1 : Age 1**	-0.0226	0.0695	-0.32	0.7454	0.9777	**ns**

Post-Hoc Pairwise Comparisons (EMMeans & Odds Ratios)

5A. Estimated Marginal Means for Block Duration (Back-transformed to Probability scale)

Block Duration (ms)	Probability	Std. Error	Df	). 95% LCL	p. 95% UCL
**20**	0.9073	0.0119	Inf	0.8811	0.9282
**40**	0.8459	0.0164	Inf	0.8109	0.8755
**80**	0.4526	0.0282	Inf	0.3982	0.5082
**160**	0.0800	0.0094	Inf	0.0635	0.1004
**320**	0.0162	0.0028	Inf	0.0115	0.0227

5B. Main Effect Pairwise Comparisons for Block Duration (Tukey Adjusted)

Contrast	Odds Ratio	Std. Error	z.ratio	p.value	Significance
**20ms / 40ms**	1.78	0.22	4.799	< 0.0001	***
**20ms / 80ms**	11.84	1.31	22.295	< 0.0001	***
**20ms / 160ms**	112.57	14.40	36.926	< 0.0001	***
**20ms / 320ms**	596.34	106.00	35.832	< 0.0001	***
**40ms / 80ms**	6.64	0.59	21.155	< 0.0001	***
**40ms / 160ms**	63.13	6.89	37.956	< 0.0001	***
**40ms / 320ms**	334.44	55.30	35.173	< 0.0001	***
**80ms / 160ms**	9.51	0.86	24.986	< 0.0001	***
**80ms / 320ms**	50.36	7.67	25.744	< 0.0001	***
**160ms / 320ms**	5.30	0.84	10.509	< 0.0001	***

5C. Pairwise Comparisons of Block Duration WITHIN Word Familiarity Levels

Familiarity Level	Contrast	)dds Ratio	Std. Error	z.ratio	p.value	Significance
**Word Familiarity 1 (High)**	20ms / 40ms	1.95	0.43	3.023	0.0211	*
**Word Familiarity 1 (High)**	20ms / 80ms	21.50	4.21	15.677	< 0.0001	***
**Word Familiarity 1 (High)**	20ms / 160ms	314.02	66.40	27.206	< 0.0001	***
**Word Familiarity 1 (High)**	20ms / 320ms	2,161.57	603.00	27.541	< 0.0001	***
**Word Familiarity 1 (High)**	40ms / 80ms	11.02	1.65	16.038	< 0.0001	***
**Word Familiarity 1 (High)**	40ms / 160ms	160.97	27.20	30.074	< 0.0001	***
**Word Familiarity 1 (High)**	40ms / 320ms	1,108.02	275.00	28.264	< 0.0001	***
**Word Familiarity 1 (High)**	80ms / 160ms	14.61	1.81	21.641	< 0.0001	***

**Word Familiarity 1 (High)**	80ms / 320ms	100.54	22.00	21.117	< 0.0001	***
**Word Familiarity 1 (High)**	160ms / 320ms	6.88	1.54	8.638	< 0.0001	***
**Word Familiarity 2 (Low)**	20ms / 40ms	1.63	0.16	5.095	< 0.0001	***
**Word Familiarity 2 (Low)**	20ms / 80ms	6.52	0.65	18.793	< 0.0001	***
**Word Familiarity 2 (Low)**	20ms / 160ms	40.35	5.40	27.624	< 0.0001	***
**Word Familiarity 2 (Low)**	20ms / 320ms	164.52	35.00	23.967	< 0.0001	***
**Word Familiarity 2 (Low)**	40ms / 80ms	4.00	0.38	14.563	< 0.0001	***
**Word Familiarity 2 (Low)**	40ms / 160ms	24.76	3.22	24.683	< 0.0001	***
**Word Familiarity 2 (Low)**	40ms / 320ms	100.94	21.20	21.927	< 0.0001	***
**Word Familiarity 2 (Low)**	80ms / 160ms	6.19	0.80	14.160	< 0.0001	***
**Word Familiarity 2 (Low)**	80ms / 320ms	25.23	5.28	15.419	< 0.0001	***
**Word Familiarity 2 (Low)**	160ms / 320ms	4.08	0.92	6.242	< 0.0001	***

5D. Pairwise Comparisons of Block Duration WITHIN Age Groups (Tukey Adjusted)

Age Group	Contrast	Odds Ratio	Std. Error	z.ratio	p.value	Significance
**Age Group 1**	20ms / 40ms	1.51	0.22	2.768	0.0438	*
**Age Group 1**	20ms / 80ms	14.41	2.11	18.234	< 0.0001	***
**Age Group 1**	20ms / 160ms	172.93	30.10	29.541	< 0.0001	***
**Age Group 1**	20ms / 320ms	1,121.34	281.00	28.112	< 0.0001	***
**Age Group 1**	40ms / 80ms	9.54	1.18	18.102	< 0.0001	***
**Age Group 1**	40ms / 160ms	114.52	17.20	31.025	< 0.0001	***
**Age Group 1**	40ms / 320ms	742.61	169.00	29.314	< 0.0001	***
**Age Group 1**	80ms / 160ms	12.01	1.48	20.215	< 0.0001	***
**Age Group 1**	80ms / 320ms	77.84	15.60	21.841	< 0.0001	***
**Age Group 1**	160ms / 320ms	6.48	1.29	9.412	< 0.0001	***
**Age Group 2**	20ms / 40ms	2.11	0.29	5.421	< 0.0001	***
**Age Group 2**	20ms / 80ms	9.73	1.25	17.412	< 0.0001	***
**Age Group 2**	20ms / 160ms	73.28	11.20	28.114	< 0.0001	***
**Age Group 2**	20ms / 320ms	317.15	68.40	26.415	< 0.0001	***
**Age Group 2**	40ms / 80ms	4.61	0.51	13.841	< 0.0001	***
**Age Group 2**	40ms / 160ms	34.73	4.95	25.102	< 0.0001	***
**Age Group 2**	40ms / 320ms	150.31	31.80	23.811	< 0.0001	***
**Age Group 2**	80ms / 160ms	7.53	0.94	16.214	< 0.0001	***

**Age Group 2**	80ms / 320ms	32.60	6.72	17.105	< 0.0001	***
**Age Group 2**	160ms / 320ms	4.33	0.81	7.854	< 0.0001	***

5E. Pairwise Comparisons for Word Familiarity × Age Interaction

5E.1. Word Familiarity Effect WITHIN Each Age Group (Tukey/Sidak Adjusted)

Age Group	Contrast (Familiarity)	Odds Ratio	Std. Error	z.ratio	p.value	Significance
**Age Group 1**	High Familiarity / Low Familiarity	5.00	0.45	17.652	< 0.0001	***
**Age Group 2**	High Familiarity / Low Familiarity	2.68	0.24	11.025	< 0.0001	***

5E.2. Full Cross-Group Pairwise Comparisons (Tukey Adjusted across all 4 cells)

Group 1 (Fam, Age)	Group 2 (Fam, Age)	Odds Ratio	Std. Error	z.ratio	p.value	Significance
**High Fam, Age 1**	Low Fam, Age 1	5.00	0.45	17.652	< 0.0001	***
**High Fam, Age 1**	High Fam, Age 2	2.61	0.44	5.728	< 0.0001	***
**High Fam, Age 1**	Low Fam, Age 2	7.01	1.15	11.841	< 0.0001	***
**Low Fam, Age 1**	High Fam, Age 2	0.52	0.09	-3.854	< 0.0011	***
**Low Fam, Age 1**	Low Fam, Age 2	1.40	0.23	2.025	0.1782	ns
**High Fam, Age 2**	Low Fam, Age 2	2.68	0.24	11.025	< 0.0001	***

Random Effects Variance Structure

Variance components, standard deviations, and correlations for grouping factors

Group	Name / Parameter	Variance	Std.Dev.	Corr
**Participant ID**	(Intercept)	0.3574	0.5978	-
**Participant ID**	Morae_number_c	0.0850	0.2915	-0.01
**Word ID**	(Intercept)	0.3042	0.5515	-

Supplementary Material S2

Results of the GLMM analysis as under Supplementary Material S1, with the full data set including the original-word data.

1. Fixed Effects (Type III Wald Chi-Square Tests)

Fixed Effect / Interaction Term	Chisq (χ^2)	Degrees of Freedom (Df)	p-value	Significance
**Block duration**	2,974.7039	5	**2.20E-16**	***
**Word Familiarity**	164.7135	1	**2.20E-16**	***
**Age**	14.8645	1	**1.16E-04**	***
**Trial_c (Trial Order)**	14.0722	1	**1.76E-04**	***
Morae_number_c (Mora Cou	0.5235	1	0.4694	ns
**Block duration × Word Familiarity**	143.8688	5	**2.20E-16**	***
**Block duration × Age**	35.4823	5	**1.21E-06**	***
Word Familiarity × Age	2.8298	1	0.0925	. (Trend)
Block duration × Word Familiarity × Age	10.5100	5	0.0620	. (Trend)

2. Random Effects Variance Components

Group (Random Effect)	Effect Term	Variance (σ^2)	Std. Dev. (σ)
**Word ID (Item)**	Intercept	0.1248	0.3533
**Participant ID**	Intercept	0.4512	0.6717
**Participant ID**	Slope: Morae_r	0.0315	0.1775
**Residual Dispersion**	Beta-Binomial	-	8.4210

Beta-Binomial GLMM Analysis: Full Pairwise Comparisons & Exact p-values

Model: model_full (glmmTMB) | Response: cbind(Moraecorrect, Moraefail) | Adjustments: Tukey / Sidak

1. Main Effects Pairwise Comparisons

Factor / Effect	Contrast / Comparison	Estimate (Log-Odds)	SE	Odds Ratio	z-ratio	p-value	Significance
Word Familiarity	Fam 1 (High) vs. Fam 2 (Low)	1.41	0.1100	4.10	12.83	1.000E-08	p < .0001 ***
Age Group	Age 3 vs. Age 4	0.6582	0.1707	1.93	3.86	0.0001	p < .001 ***
Block Duration	0 ms vs. 20 ms	1.42	0.2312	4.15	6.16	1.000E-07	p < .0001 ***
Block Duration	20 ms vs. 40 ms	0.5123	0.1411	1.67	3.63	0.0038	p < .01 **
Block Duration	40 ms vs. 80 ms	1.89	0.1245	6.63	15.20	1.000E-08	p < .0001 ***
Block Duration	80 ms vs. 160 ms	2.23	0.1420	9.31	15.71	1.000E-08	p < .0001 ***
Block Duration	160 ms vs. 320 ms	1.63	0.2810	5.11	5.81	5.000E-07	p < .0001 ***

2. Pairwise Comparisons: Block Duration x Word Familiarity

Word Familiarity	Contrast (Block Duration Comparison)	Estimate (Log-Odds)	SE	Odds Ratio	z-ratio	p-value (Tukey)	Significance
Fam 1 (High)	0 ms / 20 ms	1.29	0.3426	3.62	3.76	0.0024	p < .01 **
Fam 1 (High)	0 ms / 40 ms	1.96	0.3189	7.07	6.13	0.0001	p < .001 ***
Fam 1 (High)	0 ms / 80 ms	4.36	0.3022	78.50	14.44	1.000E-08	p < .0001 ***
Fam 1 (High)	0 ms / 160 ms	7.05	0.3127	1148.10	22.53	1.000E-08	p < .0001 ***
Fam 1 (High)	0 ms / 320 ms	8.98	0.3619	7916.42	24.81	1.000E-08	p < .0001 ***
Fam 1 (High)	20 ms / 40 ms	0.6693	0.2209	1.95	3.03	0.0295	p < .05 *
Fam 1 (High)	40 ms / 80 ms	2.41	0.1493	11.10	16.12	1.000E-08	p < .0001 ***
Fam 1 (High)	80 ms / 160 ms	2.68	0.1232	14.63	21.77	1.000E-08	p < .0001 ***
Fam 1 (High)	160 ms / 320 ms	1.93	0.2231	6.90	8.65	1.000E-08	p < .0001 ***
Fam 2 (Low)	0 ms / 20 ms	1.58	0.1532	4.84	10.29	1.000E-08	p < .0001 ***
Fam 2 (Low)	0 ms / 40 ms	2.06	0.1511	7.88	13.66	1.000E-08	p < .0001 ***
Fam 2 (Low)	0 ms / 80 ms	3.46	0.1543	31.72	22.41	1.000E-08	p < .0001 ***
Fam 2 (Low)	0 ms / 160 ms	5.28	0.1785	196.57	29.59	1.000E-08	p < .0001 ***
Fam 2 (Low)	0 ms / 320 ms	6.69	0.2436	804.14	27.47	1.000E-08	p < .0001 ***
Fam 2 (Low)	20 ms / 40 ms	0.4873	0.0956	1.63	5.10	5.000E-06	p < .0001 ***
Fam 2 (Low)	40 ms / 80 ms	1.39	0.0948	4.03	14.69	1.000E-08	p < .0001 ***
Fam 2 (Low)	80 ms / 160 ms	1.82	0.1284	6.20	14.20	1.000E-08	p < .0001 ***
Fam 2 (Low)	160 ms / 320 ms	1.41	0.2251	4.09	6.26	1.000E-07	p < .0001 ***

3. Pairwise Comparisons: Block Duration x Age Group

Age Group	Contrast (Block Duration Comparison)	Estimate (Log-Odds)	SE	Odds Ratio	z-ratio	p-value (Tukey)	Significance
Age 3	0 ms / 20 ms	1.74	0.2219	5.67	7.82	1.000E-08	p < .0001 ***
Age 3	0 ms / 40 ms	2.42	0.2169	11.23	11.15	1.000E-08	p < .0001 ***
Age 3	0 ms / 80 ms	4.22	0.2180	67.80	19.34	1.000E-08	p < .0001 ***
Age 3	20 ms / 40 ms	0.6834	0.1400	1.98	4.88	2.000E-05	p < .0001 ***
Age 3	40 ms / 80 ms	1.80	0.1190	6.04	15.11	1.000E-08	p < .0001 ***
Age 3	80 ms / 160 ms	2.51	0.1322	12.35	19.01	1.000E-08	p < .0001 ***
Age 3	160 ms / 320 ms	2.03	0.2297	7.62	8.84	1.000E-08	p < .0001 ***
Age 4	0 ms / 20 ms	1.19	0.1976	3.28	6.01	1.000E-07	p < .0001 ***
Age 4	0 ms / 40 ms	1.48	0.2018	4.38	7.32	1.000E-08	p < .0001 ***
Age 4	0 ms / 80 ms	3.35	0.2034	28.54	16.48	1.000E-08	p < .0001 ***
Age 4	20 ms / 40 ms	0.2893	0.1327	1.34	2.18	0.2312	Not Significant
Age 4	40 ms / 80 ms	1.87	0.1259	6.52	14.89	1.000E-08	p < .0001 ***
Age 4	80 ms / 160 ms	1.99	0.1433	7.34	13.91	1.000E-08	p < .0001 ***
Age 4	160 ms / 320 ms	1.33	0.2504	3.78	5.31	1.000E-06	p < .0001 ***

4. Pairwise Comparisons: Word Familiarity x Age Group

Age Group	Contrast (Familiarity Comparison)	Estimate (Log-Odds)	SE	Odds Ratio	z-ratio	p-value	Significance
Age 3	Fam 1 (High) vs. Fam 2 (Low)	1.44	0.0895	4.23	16.11	1.000E-08	p < .0001 ***
Age 4	Fam 1 (High) vs. Fam 2 (Low)	1.41	0.1015	4.08	13.85	1.000E-08	p < .0001 ***

Supplementary Material S3

GLMM with the intelligibility data from the elderly participants and four-frequency PTA (renewed) combined across ears as a continuous predictor.

Model Description:
To better evaluate the effects of hearing loss on intelligibility in elderly listeners, a generalized linear mixed model (GLMM) was fitted with a beta-binomial distribution and a logit link function (using glmmTMB package in R). The model included the same fixed main effects, control covariates, and random intercepts as described in the paper for the “full model” (P9), yet instead of “Age (group)” continuous standardized Hearing Level (HL) was used, i.e., individual four-frequency PTA (500, 1000, 2000, 4000 Hz) across both ears. Overdispersion across block duration was modeled via a custom dispersion formula. Type III Wald chi-square tests were evaluated using orthogonal sum contrasts.

Results:
Type III Wald tests revealed a significant main effect of HL [χ^2 (1)=27.12,p<.001], indicating that higher HL was associated with lower overall morae intelligibility. Similar to the results of the model described in the paper (e.g., P10, Table 1), significant main effects were also observed for block duration [χ^2 (4)=1173.90,p<.001], word familiarity [χ^2 (1)=65.64,p<.001], and trial number [χ^2 (1)=11.62,p<.001]. Morae number was not a significant predictor [χ^2 (1)=0.13,p=.722].
The negative effect of hearing loss was moderated by experimental conditions. The interaction between block duration and HL was statistically significant [χ^2 (4)=21.85,p<.001], as was the interaction between word familiarity and HL [χ^2 (1)=4.74,p=.029]. The three-way interaction between block duration, word familiarity, and HL was not statistically significant [χ^2 (4)=4.47,p=.346]. Additionally, a strong two-way interaction emerged between block duration and word familiarity [χ^2 (4)=83.42,p<.001].

To probe the block duration × HL and the word familiarity × HL interactions, simple slopes (β) of standardized HL were evaluated across conditions on the log-odds scale.
For high-familiarity words, HL had a strong, statistically significant negative impact on morae intelligibility at mosaic block durations of:

20 ms (β=-0.85,SE=0.15,95%" CI " [-1.15,-0.54],p<.001),
40 ms (β=-1.16,SE=0.16,95%" CI " [-1.47,-0.86],p<.001),
80 ms (β=-0.83,SE=0.17,95%" CI " [-1.16,-0.50],p<.001),
and 160 ms (β=-0.52,SE=0.23,95%" CI " [-0.97,-0.08],p=.021).

At the longest temporal window of 320 ms, the effect of HL was no longer significant ($\beta=0.06, SE=0.28$, 95%" CI " [-0.48,0.60], $p=.838$).
For low-familiarity words, HL had a strong, statistically significant negative impact on morae intelligibility at mosaic block durations of:

20 ms: $\beta=-0.59, p<.001$;
40 ms: $\beta=-0.66, p<.001$;
and 80 ms: $\beta=-0.31, p=.036$).
but not at 160 ms: $\beta=-0.29, p=.177$ and 320 ms: $\beta=-0.20, p=.516$).

Conclusion:
The GLMM over the elderly listeners' data aligns with the correlation results, in that intelligibility decreased significantly as hearing loss increased for mosaic blocks up to 160 ms for high-familiarity words, and up to 80 ms for low-familiarity words.
